# Dynamic fluctuations of salivary CGRP levels during migraine attacks: association with clinical variables and phenotypic characterization

**DOI:** 10.1186/s10194-024-01772-9

**Published:** 2024-04-18

**Authors:** Alicia Alpuente, Victor J. Gallardo, Laila Asskour, Edoardo Caronna, Marta Torres-Ferrus, Patricia Pozo-Rosich

**Affiliations:** 1 Neurology Department, Headache Clinic, Vall d’Hebron University Hospital, Barcelona, Spain; 2 Headache and Neurological Pain Research Group, Vall d’Hebron Research Institute, Departament de Medicina, Universitat Autònoma de Barcelona, Barcelona, Spain

**Keywords:** Migraine, Salivary CGRP, Biomarker, Endophenotyping, Personalized medicine

## Abstract

**Background:**

Migraine is a complex neurological disorder with significant heterogeneity in its clinical presentation and molecular mechanisms. Calcitonin gene-related peptide (CGRP) has emerged as a key player in migraine pathophysiology, but challenges remain in its utilization as a biomarker. This study aimed to investigate salivary CGRP levels during migraine attacks across the frequency spectrum and explore associations with clinical variables.

**Methods:**

A prospective longitudinal pilot study was conducted, recruiting migraine patients from an outpatient headache clinic. Salivary CGRP levels were measured at interictal, onset, post-2 h of onset and end-of-attack. Using generalized linear mixed models, we explored the effect of CGRP changes over the attack in presence of depressive symptoms (DS), acute attack treatment, and after three-months of erenumab treatment. Finally, patients were classified and compared according to their CGRP phenotype.

**Results:**

A total of 44 migraine patients were included (90.9% women), with 80 migraine attacks analyzed. Salivary CGRP levels increased at the onset of migraine attacks. We observed statistically significant interactions between DS and both the linear (Est. [SE]: 19.4 [5.8], *p* = 0.001) and quadratic terms of time (-19.1 [6.0], *p* = 0.002). Additionally, a significant three-way interaction within the use of acute treated attack (linear-term: -18.5 [6.2], *p* = 0.005; quadratic-term: 19.2 [6.8], *p* = 0.005) was also found. Molecular phenotyping revealed that 72.7% (32/44) of patients presented only CGRP-dependent attacks, while 27.3% (12/44) presented non-CGRP-dependent migraine attacks. Patients with only CGRP-dependent attacks were associated with younger age, shorter disease evolution time, a higher proportion of aura, and fewer monthly headache days (*p* < 0.05). Exploratory analysis of erenumab treatment effects did not result in changes in CGRP levels during migraine attacks.

**Conclusions:**

Our study underscores the dynamic nature of migraine at a molecular level and emphasizes the importance of integrating clinical variables, such as depressive symptoms, in understanding its pathophysiology. The identification of distinct migraine subtypes based on CGRP dependence suggests potential opportunities for personalized treatment approaches.

**Supplementary Information:**

The online version contains supplementary material available at 10.1186/s10194-024-01772-9.

## Introduction

Migraine is a neurological disorder characterized by cyclic paroxysmal multiphase attacks of head pain and a myriad of neurological symptoms [1]. It is a prominent cause of disability due to its profound impact on individuals. The frequent occurrence of comorbid conditions such as depression and anxiety, which themselves rank among the top ten causes of global disability, further consolidates migraine’s position as one of the most disabling disorders worlwide [2, 3].

Calcitonin gene-related peptide (CGRP) is a neuropeptide that plays a clear role in migraine pathophysiology, including neurogenic inflammation of trigeminal nerve fibers, dural vasodilation, and nociceptive transmission in the peripheral and central nervous system [4, 5]. While validated biomarkers for migraine are lacking [6], the discovery of CGRP’s implication in migraine pathophysiology, its release during acute migraine attacks [7, 8], evidence from human models demonstrating CGRP’s ability to trigger migraine attacks in susceptible patients [9], and the fact that blocking CGRP represented a clinically meaningful treatment for migraine [10], collectively position CGRP as the most promising candidate. Up to now, four monoclonal antibodies (mAbs) have been developed for migraine treatment: three target the CGRP ligand (fremanezumab, galcanezumab, and eptinezumab), and one targets the CGRP receptor (erenumab).

However, quantifying CGRP is challenging, and researchers have encountered numerous methodological difficulties [11], with no validated standardized method. Nonetheless, experimental studies on CGRP are crucial in order to phenotype patients from a molecular perspective and contribute to a pathophysiological-driven classification in migraine. Thus, several studies have measured CGRP in different substrates, in particular plasma [7, 12–16], but also in cerebrospinal fluid [17], tears [18, 19] and saliva [20–22]. Regarding the latter, we have previously demonstrated that measuring CGRP in saliva is feasible and a practical and reproducible way of measuring CGRP [23, 24].

Based on our earlier research [23, 24], we found that the CGRP salivary levels change over different migraine phases in low-frequency episodic migraine patients (LFEM). We also identified two different CGRP phenotypes related to the attack: CGRP-dependent and non-CGRP dependent attacks, each with differential symptomatology. However, the behavior of salivary CGRP levels during attacks in patients with higher headache frequency has not been previously studied, nor has the impact of this temporal profile during migraine attacks after anti-CGRP mAbs. For this reason, we aimed to investigate changes in salivary CGRP levels throughout migraine attacks in high-frequency EM (HFEM, 8–14 days/month) and chronic migraine (CM, ≥ 15 days/month), and to determine whether this dynamic fluctuation was affected after treatment with erenumab.

## Methods

### Participants and study design

This is a prospective longitudinal pilot study. Participants were recruited from the outpatient headache clinic and underwent thorough interviews conducted by a headache specialist. The recruitment period spanned from March 2018 to December 2021. We analysed the migraine attacks of patients included in the previous analysis already published [23, 24]. Adults fulfilling the criteria for migraine and CM according to the ICHD-3 were recruited [25]. Aditionnally, patients with HFEM and CM received erenumab 140 mg subcutaneously every 4 weeks according to the National Regulatory Agency [26]. No other preventive treatments were permitted.

All participants received detailed verbal, visual and written instructions for saliva collection. They were provided with appropriate materials for saliva collection at home, including pre-labeled tubes; diaries for recording sample collection times and menstrual cycle, and questionnaires for documenting migraine attack characteristics such as pain intensity and duration, accompanying symptoms and acute treatment used. Participants were instructed to manage their migraine attacks as usual, with the approval of the investigator (triptans and non-steroidal anti-inflammatory drugs (NSAIDs) were permitted). Saliva samples were collected when participants perceived the onset of their migraine attack, after 2 h, and at the conclusion of the attack. Additionally, interictal saliva samples were collected [23, 24], and the results were previously published. The interictal period was defined as having no-pain days in CM and HFEM patients and as three migraine-free days before and after a migraine day in EM patients. No migraine attacks were treated before collecting the first sample of each attack. Participants with any medical condition that could potentially alter saliva (including smoking habits, presence of chronic pain conditions such as fibromyalgia or chronic fatigue syndrome, systemic disorders such as Sjögren’s syndrome, and oral pathology) were excluded from the study.

### Clinical variables

Demographics (age and sex) and migraine characteristics were collected at baseline including aura, disease evolution time (in years), monthly headache days (MHD), monthly migraine days (MMD) and monthly acute medication intake (MAMI).

Participants also completed the Migraine Disability Assessment (MIDAS) questionnaire [27], Headache Impact Test (HIT-6) score [28], the Beck Depression Inventory (BDI-II) [29], and the Beck Anxiety Inventory [30]. Participants with ≥ 8 BAI score were classified as having anxiety symptoms and participants with ≥ 14 BDI-II score were classified as suffering from depressive symptoms (DS). All patients completed all the questionnaires using REDCap® surveys [31, 32].

### Saliva collection and CGRP quantification

The saliva collection procedure and quantification of CGRP-like immunoreactivity (referred to as CGRP hereafter) were detailed in our previous studies [23, 24]. Briefly patients collected three saliva samples during migraine attacks at home: at headache onset, after 2 h, and after 8 h. Saliva collection was conducted using the resting unstimulated whole saliva method [33] which collects saliva into sterile tubes for 5 min aiming for a minimum quantity of 3mL. After collection, baseline saliva samples were stored in participants’ freezers at -18 °C and later transported to the laboratory on ice to prevent thawing. All samples were then stored in the laboratory freezer at -80 °C. Upon CGRP extraction, samples underwent centrifugation for 20 min at 3500 rpm at -4ºC. The resulting supernatant was aliquoted into 1.5mL sterile and polypropylene Eppendorf centrifuge tubes for immediate analysis. CGRP quantification was carried out using human enzyme linked immunosorbent assay (ELISA) kits (Cusabio, detection range: 1.56–100 pg/ml, minimal detectable dose: 0.39 pg/ml). Duplicate measurements were performed for each sample. CGRP concentrations were determined from calibration curves using a 4PL fitting (log scale concentration) as implemented in the Analysis software Gen5 resulting in a fit with R2 > 0.99 in every case. The final CGRP level of each sample was calculated as the average of the two measurements. Internal validation of the test was conducted and CGRP concentrations from the immunoassay procedure were corrected by inter and intra-assay coefficients of variability for each ELISA plate.

### Statistical analysis

This exploratory analysis is a secondary pre-planned analysis of previously collected data [23, 24]. Nominal variables (sex, aura and the presence of anxiety or depression) were reported as frequencies (percentages) while median and interquartile range (IQR) were reported for quantitative variables (age, disease evolution time, MHD, MMD, monthly acute medication intake, MIDAS and HIT-6). Normality assumption of quantitative variables was checked through visual methods (Q-Q plots) and normality tests (Shapiro-Wilk test).

Statistical significance between CGRP phenotypes (Non-CGRP dependent vs. CGRP dependent migraine attacks) was assessed by Fisher’s exact test when comparing categorical variables (accompanying symptoms rate, sex, aura, anxiety and DS), independent t-test for age, pain intensity, MHD and HIT-6 was used for comparing continuous variables or Wilcoxon rank-sum test for the other quantitative variables that did not follow any normality assumption. After 12 weeks of treatment with erenumab, statistical significance pre-post treatment for continuous data was performed with paired t-test or paired Wilcoxon signed-rank test, considering data distribution, and McNemar’s test was performed for categorical data.

To fulfil the objectives of this secondary analysis, two different multivariate generalized mixed-effect regression models (GLMMs) were estimated in order to study whether the change in salivary CGRP quantification over the different migraine phases (interictal, onset, after 2 h and end of the attack) remained significant after the inclusion of new migraine attacks from participants with HFEM and CM; and [2] whether this change was associated with erenumab after 12-week of active treatment period.

As reported previously [23], GLMM are powerful and flexible statistical models that are particularly well-suited for analyzing longitudinal data, especially when there is no independence in data (different salivary CGRP measurements from the same patient over the migraine attack) and to account for patient-specific variability that need to be considered as random effects. Morever, ELISA plates were also considered as random effects. All independent variables were scaled and centered before model fitting and only random intercepts per participant were implemented.

Full models were fitted using R package glmmTMB v.1.1.7 and variance inflation factors (VIFs) for all the parameters were computed in order to estimate how much the variance of an estimated regression coefficient is inflated due to correlated variables so that we could avoid an overfitting problem in the final models. Model diagnostic plots (residual QQ plot, residuals vs. fitted quantile plot and overdispersion test) was performed using R package DHARMa v.0.4.6. The analysis of deviance table of model’s main effect was performed and effect plots were plotted using the R sjPlot package v.2.8.14.

All statistical analysis were conducted in R v4.3.1 and *p*-values < 0.05 were considered as statistically significant and are reported for a two-tailed test.

### Data availability

Data not published within this article will be made available by request from any qualified investigator.

### Standard protocol approvals and patient consent

All patients voluntarily signed consent forms for their participation in the study. Approval for this study was obtained from the Vall d’Hebron Ethics Committee PR(AG)590/2021. Approval for the first study was obtained from the Vall d’Hebron Ethics Committee PR(IR)292/2017. All participants gave their consent for data collection.

## Results

### Descriptive

**Fig. 1:**
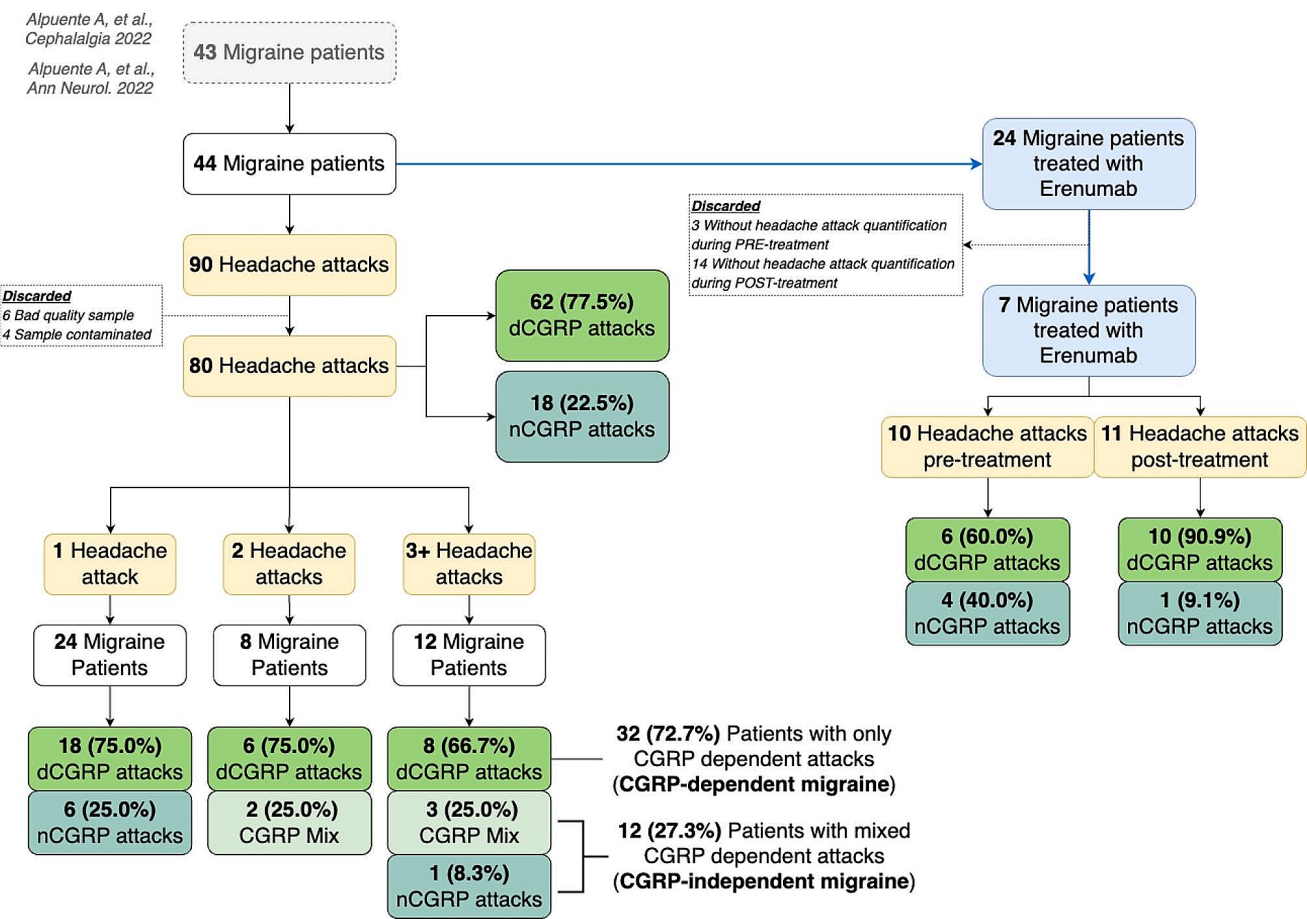
Participants and headache attacks flowchart. dCGRP: CGRP dependent attack; nCGRP: non-CGRP dependent attack

A total of 44 migraine patients were enrolled in the study, resulting in the collection of data from 90 migraine attacks initially. Following the exclusion of samples of poor quality, data from 80 migraine attacks were ultimately analyzed. Figure 1 illustrates the flowchart depicting patient enrollment and migraine attack inclusion.

Of the 44 patients included, 40 (90.9%) were women, with a median (IQR) age of 35.5 (26.5, 46.2) years. The median headache frequency at baseline was 10.5 (7.0, 18.2) days/month, and the median migraine frequency 6.0 (3.0, 10.2) days/month. DS were present in 22.7% (10/44) of patients at baseline. Additional information regarding comorbidities and migraine characteristics can be found in Table 1.

**Table 1:** Demographics, comorbidities and migraine characteristics at baseline

Variables	Patients (*n* = 44)
**Demographics**	
Age, y	35.5 (26.5, 46.2)
Female	40 (90.9%)
**Comorbidities**	
Anxiety	27 (61.4%)
Depression	10 (22.7%)
**Migraine characteristics**	
Duration of migraine disease, y	18.5 (10.0, 28.0)
Aura	22 (50.0%)
Headache frequency (MHD), d/mo	10.5 (7.0, 18.2)
Migraine frequency (MMD), d/mo	6.0 (3.0, 10.2)
Acute medication frequency, d/mo	8.0 (6.0, 14.2)
**Migraine-related burden**	
MIDAS, score	26.5 (14.2, 58.5)
HIT-6, score	64.5 (61.8, 67.0)

Continuous data is represented in median (IQR) and categorical data in % (n). IQR: interquartile range; y: years, MHD monthly headache days; MMD: monthly migraine days; d/mo: days/month; MIDAS: migraine disability assessment; HIT-6: headache impact test

Anxiety was considered when patients had ≥ 8 BAI score and depression, ≥ 14 BDI-II score

### Salivary CGRP levels during migraine attacks

Variables associated with changes in CGRP levels included the main effect of the presence of DS, which was linked to a statistically significant increase in salivary CGRP levels (*p* = 0.021). Additionally, two-way interactions were observed between DS and attack evolution (both linear, *p* = 0.001, and quadratic, *p* = 0.002), indicating a positive correlation. Specifically, in the presence of DS, CGRP levels were increased, particularly during the onset of the attack (quadratic effect).

Another significant finding was a two-way interaction between an acutely treated attacks and the quadratic term of CGRP evolution (*p* = 0.046), suggesting that when attacks were treated, CGRP levels decreased at the end of the attack, in contrast to untreated attacks, where CGRP levels showed a sustained increase (linear trend). The model’s coefficients and significance are presented in Table 2.

**Table 2:** Estimated coefficients, coefficients’ standard error (SE), 95% CI and *p*-values for salivary CGRP quantification of the fitted GLMM during a migraine attack characterization

Independent Variables^†^	Estimate	SE	95% CI	*P*-value^‡^
*(Intercept)*	10.55	0.695	9.19–11.91	**< 0.001**
**Main effects**				
Age, y	-0.306	0.527	-1.34–0.727	0.562
Depressive symptoms (at baseline)				
No (Ref.) Yes	- 4.11	- 1.78	- 0.611–7.61	- **0.021**
Treated attack (acute medication)				
No (Ref.) Yes	- 0.087	- 0.659	- -1.21–1.38	- 0.895
Attack evolution				
Linear (L) Quadratic (Q)	-0.172 1.62	1.88 1.88	-3.85–3.51 -2.07–5.32	0.927 0.389
**Two-way interactions**				
DS × AM	-1.93	1.69	-5.24–1.38	0.253
DS × Time [L]	19.35	5.81	7.95–30.74	**0.001**
DS × Time [Q]	-19.06	6.01	-30.85– -7.28	**0.002**
Treated attack × Attack evolution [L]	3.68	2.49	-1.20–8.55	0.139
Treated attack × Attack evolution [Q]	-5.03	2.53	-9.99– -0.079	**0.046**
**Three-way interactions**				
DS × Treated attack × Attack evolution [L]	-18.45	6.23	-31.44– -5.47	**0.005**
DS × Treated attack × Attack evolution [Q]	19.19	6.80	5.85–32.53	**0.005**

SE: Standard Error; CI: confidence interval; L: linear; Q: quadratic; DS: presence of depressive symptoms; ‘×’ symbol indicates interaction between variables

**Bold** font indicates statistically significant variables. †Continuous independent variables were rescaled to a z-score metric (mean = 0, SD = 1) in the mixed model; ‡Statistical significance assessed the analysis of Deviance in each model (Type III Wald chi-square test)

**Fig. 2:**
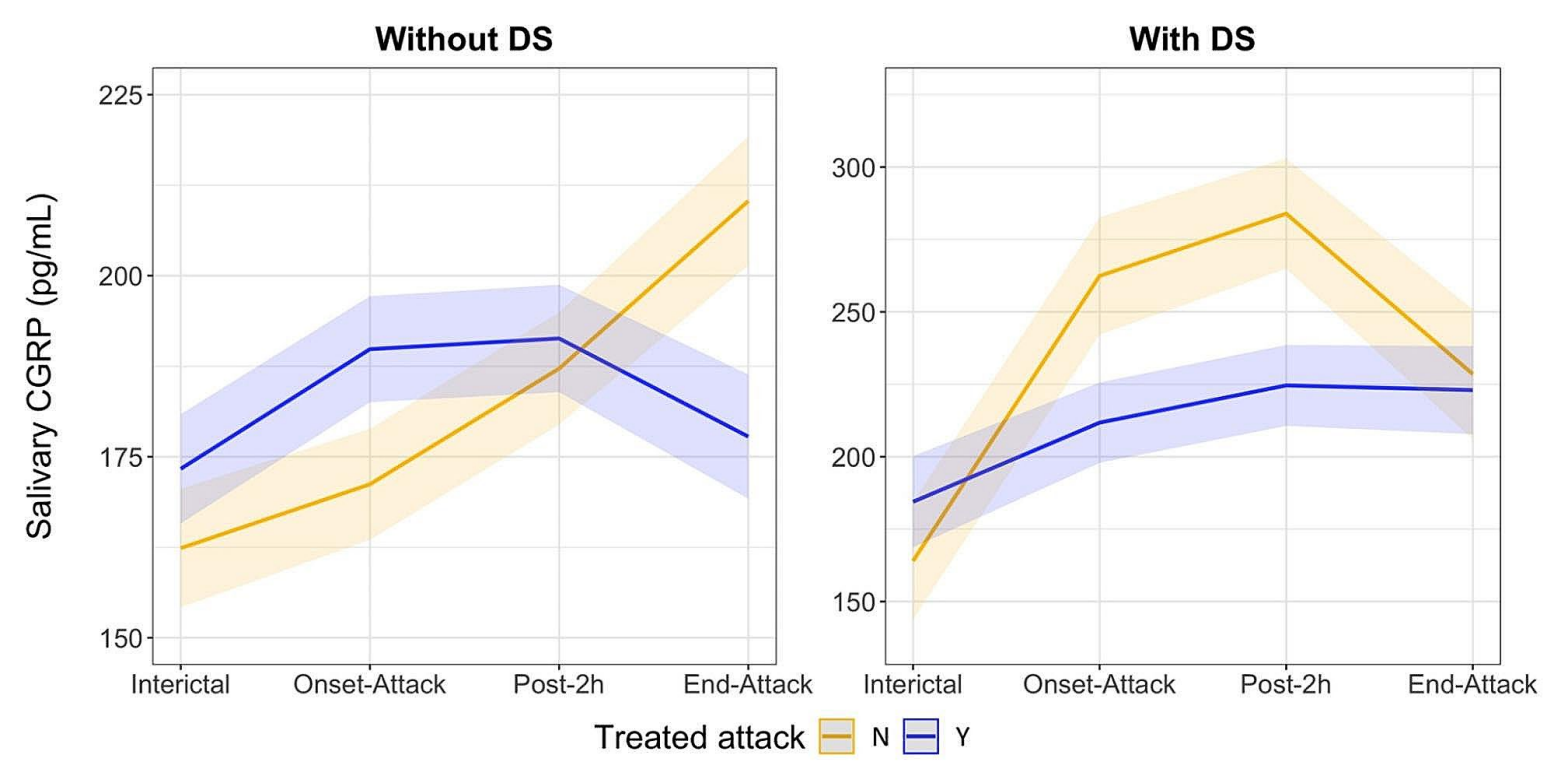
Salivary CGRP quantification (pg/mL) during a migraine attack according the presence of depressive symptoms at baseline and acute treatment. All predictors were rescaled to a z-score metric (mean = 0, SD = 1) in the prediction model. The shadow zone represents the 95% confidence levels of the GLMM estimation

Furthermore, a three-way interaction between the presence of DS, acutely treated attacks, and CGRP evolution was also statistically significant (*p* = 0.005 for both linear and quadratic terms). The effect plot of this three-way interaction is shown in Fig. 2, revealing that in the presence of DS, salivary CGRP levels were more resistant to reduction after acute treatment. Conversely, when attacks were not treated, patients with DS exhibited higher CGRP levels during the onset and after 2 h, while in patients without DS, this increase was more gradual.

### Molecular endophenotype

Furthermore, we characterized migraine attacks as either CGRP dependent (dCGRP) or non-CGRP-dependent (nCGRP), based on the presence or absence of an increase in salivary CGRP between interical and the onset of the attack, as previously described [24]. Out of the 80 attacks analyzed, 77.5% (62/80) were classified as dCGRP, while 22.5% (18/80) were classified as nCGRP (Fig. 1). Statistically significant variables associated with dCGRP included acutely treated attack (*p* = 0.003), allodynia (*p* = 0.046), swelling (*p* = 0.006), cervical pain (*p* = 0.003), and ocular pain (*p* = 0.001) (Table 3).

**Table 3:** Clinical characteristics associated with CGRP dependent and non-CGRP dependent headache attacks

Variables	Non-CGRP dependent (*n* = 18)	CGRP dependent (*n* = 62)	Missing	*P* value
**Attack characteristics**				
Aura	5 (35.7%)	10 (20.4%)	17	0.291^†^
Pain intensity, (0–10)	6.0 (5.0, 7.0)	6.0 (5.0, 8.0)	22	0.584^‡^
Treated attack at 2 h	10 (100.0%)	24 (50.0%)	22	**0.003** ^**†**^
**Accompanying symptoms**				
Unilateral pain	6 (50.0%)	14 (30.4%)	22	0.307^†^
Throbbing pain	6 (50.0%)	9 (19.1%)	21	0.058^†^
Nausea	5 (41.7%)	27 (57.4%)	21	0.353^†^
Vomiting	0 (0.0%)	4 (8.5%)	21	0.572^†^
Photophobia	12 (100.0%)	36 (76.6%)	21	0.098^†^
Phonophobia	10 (83.3%)	31 (66.0%)	21	0.311^†^
Osmophobia	2 (16.7%)	10 (21.3%)	21	> 0.999^†^
Allodynia	3 (25.0%)	28 (59.6%)	21	**0.046** ^**†**^
Dizziness	4 (33.3%)	17 (36.2%)	21	0.734^†^
Swelling	5 (41.7%)	3 (6.4%)	21	**0.006** ^**†**^
Cervical pain	7 (58.3%)	10 (21.3%)	21	**0.027** ^**†**^
Ocular pain	7 (58.3%)	5 (10.6%)	21	**0.001** ^**†**^

Continuous data is represented in median (IQR) and categorical data in % (n). IQR: interquartile range

**Bold** font indicates statistically significant variables

†Statistical significance assessed with Fisher’s exact test

‡Statistical significance assessed with unpaired t-test

Finally, we phenotyped patients considering their ratio of dCGRP/nCGRP: when all their migraine attacks were dCGRP, we diagnosed patients with CGRP-dependent migraine, whereas if they had non-CGRP dependent attacks or mix of both (dCGRP and nCGRP), we diagnosed them as CGRP-independent migraine. Among the 44 patients included, 72.7% were phenotyped with CGRP-dependent migraine (Fig. 1). When comparing these two migraine phenotypes, we found that younger patients (*p* = 0.013), lower disease evolution time (*p* = 0.040), higher proportion of aura (*p* = 0.044), and lower MHD (*p* = 0.010) were associated with strictly CGRP-dependent migraine (Table 4).

**Table 4:** Clinical characteristics associated with migraine’s phenotype

Variables	CGRP-Independent migraine (*n* = 7)	CGRP-Dependent migraine (*n* = 32)	*P* value
**Demographics**			
Age, y	47.0 (41.0, 51.5)	28.0 (23.8, 38.2)	**0.013** ^**†**^
Female	7 (100.0%)	28 (87.5%)	> 0.999^*^
**Comorbidities**			
Anxiety	5 (71.4%)	20 (62.5%)	> 0.999^*^
Depression	1 (14.3%)	5 (15.6%)	> 0.999^*^
**Migraine characteristics**			
Duration of migraine disease, y	27.0 (22.0, 34.5)	15.5 (8.0, 25.5)	**0.040** ^**‡**^
Aura	1 (14.3%)	19 (59.4%)	**0.044** ^*^
Headache frequency (MHD), d/mo	18.0 (14.0, 23.0)	9.5 (6.0, 16.0)	**0.010** ^**†**^
Migraine frequency (MMD), d/mo	8.0 (6.0, 16.0)	6.0 (3.0, 10.0)	0.108^‡^
Acute medication frequency, d/mo	10.0 (8.5, 16.0)	8.0 (6.0, 12.2)	0.136^‡^
**Migraine-related clinical burden**			
Disability (MIDAS), score	31.0 (19.0, 69.5)	22.5 (10.0, 56.5)	0.410^‡^
Headache-related impact (HIT-6), score	66.0 (66.0, 67.0)	64.0 (61.0, 68.0)	0.335^†^

Continuous data is represented in median (IQR) and categorical data in % (n). IQR: interquartile range; y: years, MHD monthly headache days; MMD: monthly migraine days; d/mo: days/month; MIDAS: migraine disability assessment; HIT-6: headache impact test

Anxiety was considered when patients had ≥ 8 BAI score and depression, ≥ 14 BDI-II score

**Bold** font indicates statistically significant variables

*Statistical significance assessed with the Fisher’s exact test

†Statistical significance assessed with unpaired t-test

‡Statistical significance assessed with unpaired Mann-Whitney U test

### Exploratory analysis: migraine attacks after anti-CGRP targeted therapy

Out of the initially recruited 44 patients, 24 received treatment with erenumab 140 mg. Among this group, salivary CGRP levels during migraine attacks were collected from 7 patients both before and after 12 weeks of treatment, resulting in a total of 10 headache attacks before treatment and 11 after treatment (Fig. 1).

We readjusted the previous GLMM model to identify differences in salivary CGRP levels during headache attack before and after treatment. The model’s estimations are presented in Supplementary Table 1. We continued to find a main effect of DS on salivary CGRP levels (*p* < 0.001), but we also discovered a statistically significant two-way interaction (*p* < 0.001) between the presence of DS at baseline and preventive treatment period (baseline vs. follow-up, *p* = 0.003). This interaction suggests that at baseline, CGRP levels were higher in patients with DS but after three months of treatment, no differences were found in the salivary CGRP pattern of headache attacks between patients with and without DS (Supplementary Fig. 1). However, the time-evolution trend was not statistically significant (linear *p* = 0.743, quadratic *p* = 0.811), indicating no increase in CGRP during the migraine attack before and after treatment.

Finally, we compared the proportion of dCGRP and nCGRP before and after 12 weeks of treatment, but this did not reach the statistical significance level (baseline: 60.0% (6/10) dCGRP vs. follow-up: 90.9% (10/11) dCGRP, *p* = 0.077).

## Discussion

In this study, we investigated salivary CGRP levels throughout migraine attacks across patients of the entire frequency spectrum. We found dynamic fluctuations in CGRP levels during different phases of the attacks, influenced notably by the presence of DS and acute treatment. Furthermore, our study underscores the molecular heterogeneity of migraine, highlighting distinct phenotypes among migraine patients.

Our results highlight the dynamic nature of migraine at the molecular level. While clinical phases of migraine attack are well-established [34], our study demonstrates that molecular changes, particularly in CGRP levels, also vary throughout the course of a migraine attack. Notably, DS and acute significantly affected CGRP levels during these attacks, suggesting broader factors shaping migraine pathophysiology. The role of DS in migraine is of particular importance. The association between depression and migraine is well-established both clinically [35–38] and genetically [39, 40]. At molecular level, we have increasing evidence about the link between CGRP and depression. Altered levels of CGRP-LI in animal models [41, 42] and in the cerebrospinal fluid of depressed patients has been reported [43], suggesting that CGRP may be involved in the pathophysiology and/or be a trait marker of major depressive disorder. Increased brain levels of CGRP have been found in a well-established rat model of depression and, interestingly, antidepressants did not have effect on the brain level of this peptide [41]. Furthermore, a recent study showed that DS improve with anti-CGRP mAbs, regardless of improvement in classical outcomes [44]. Previous results of our group showed that the increase of MHD was associated to an increase of the CGRP levels at baseline and that this increase was even higher in presence of DS [23]. Our findings contribute to this body of knowledge by demonstrating a link between CGRP levels and DS during migraine attacks.

Our study also confirms previous findings regarding the effects of acute treatment on CGRP levels during migraine attacks [45]. Treatment with triptans has been shown to reduce CGRP levels during migraine attacks in classical studies [8, 21]. Our results extend this understanding by demonstrating that this reduction in CGRP levels occurs not only in patients with LFEM but also in those with HFEM and CM. However, the presence of DS appears to influence the efficacy of acute treatment, with CGRP levels remaining more resistant to reduction after treatment in patients with DS [46].

Moreover, our study provides insights into migraine molecular phenotypes, classifying attacks as either CGRP-dependent (dCGRP) or non-CGRP-dependent (nCGRP). We found associations between clinical variables and dCGRP attacks, suggesting that it is easier to distinguish migraine attacks when the headache frequency is low. However, in CM patients, it becomes very difficult to differentiate among episodes, as clinical symptoms become less differenciated [46]. In our previous study we showed that dCGRP attacks were associated with photophobia and phonophobia [24], whereas in this study, clinical variables associated with dCGRP were allodynia, ocular and cervical pain, and swelling– all indicative of the trigeminovascular system activation. Notably, younger age, shorter disease evolution time, higher aura proportion, and lower monthly headache day count were associated with strictly CGRP-dependent migraine. This suggests that CGRP may play a role in a specific subgroup of migraine patients, while other neuropeptides and mechanisms may be involved in others [6, 47–49]. Thus, CGRP may not be increased in all migraine patients, or it may be that, as for rodents, it depends on the particular individual’s gene expression for how susceptible they are to the effects of CGRP [50].

Our findings on the effects of erenumab treatment on migraine attacks are constrained by a limited number of collected attacks. We observed no differences in the CGRP variation pattern of migraine attacks between patients with and without DS after erenumab treatment. It seems that after preventive treatment with erenumab, salivary CGRP levels in patients across all migraine frequency specta converged to similar values [23] or even decrease CGRP alpha isoform in the case of galcanezumab [51]; whereas in presence of DS, CGRP levels do not reach such a convergence [23]. Furthermore, treatment with erenumab did not significantly alter the proportion of dCGRP and nCGRP migraine attacks, indicating that it may primarily affect interictal levels rather than migraine attacks themselves, increasing the threshold for having migraine attacks.

This study have several limitations. First, the study had a relatively small sample size, particularly in the analysis of migraine attacks after erenumab treatment. This limited the statistical power and generalizability of the findings, but it serves as a starting point for further investigation into the effect of erenumab in larger datasets. Secondly, patients were recruited from a single outpatient headache clinic, which may introduce selection bias and limit the representativeness of the sample to the broader migraine population, yet focusing on a single outpatient clinic allowed for consistent evaluation and potentially enhanced the homogeneity of the collected data for this pilot study. Furthermore, challenges in accurately measuring CGRP levels, including rapid degradation of the molecule, methodological issues with assay sensitivity and reliability, and the potential presence of concurrent pathologies [52, 53], may have affected the precision of the results.), may have affected the precision of the results. However, it’s worth noting that the research team has prior experience in quantifying CGRP, and internal protocols have been assessed and standardized to ensure reliable quantification and participant screenings. Lastly, the study had limited data on the effects of erenumab treatment on migraine attacks, with only a small number of attacks collected before and after treatment. This limited our ability to draw definitive conclusions about the impact of anti-CGRP receptor specific treatment on CGRP levels during attacks.

Our study also has several strengths. The study utilized a prospective longitudinal design, allowing for the collection of data over time and examination of changes in CGRP levels during migraine attacks. On the other hand, patients were comprehensively phenotyped, considering various clinical variables such as migraine frequency, presence of depressive symptoms, and response to acute treatment. This comprehensive approach, integrating molecular and clinical data, provides understanding of the heterogeneity within the migraine population. Lastly, the study conducted exploratory analyses to examine associations between CGRP levels and various clinical variables, shedding light on potential factors influencing CGRP dynamics during migraine attacks.

In conclusion, our study sheds light on the intricate interplay between salivary CGRP levels, migraine characteristics, and treatment response. Our molecular analysis replicated a significant increase in CGRP levels, particularly evident during the onset of migraine attacks, especially in patients with DS. Additionally, our patient classification, based on the ratio of CGRP-dependent (dCGRP) and non-CGRP-dependent (nCGRP) migraine attacks, provided valuable insights into distinct migraine molecular endophenotypes. The CGRP-dependent migraine endophenotype was found to be more prevalent and associated with younger age, shorter disease evolution time, higher aura prevalence, and fewer migraine headache days. In terms of the effects of erenumab on migraine attacks, our analysis revealed a notable interaction between the presence of DS and the treatment period, suggesting a normalization of CGRP patterns after three months of treatment. However, we did not observe significant changes in the proportion of dCGRP and nCGRP attacks post-treatment.

### Electronic supplementary material

Below is the link to the electronic supplementary material.

Supplementary Material 1

## Data Availability

No datasets were generated or analysed during the current study.
