# Supplementary material for: Dynamic fluctuations of salivary CGRP levels during migraine attacks: association with clinical variables and phenotypic characterization
Source: J Headache Pain. 2024 Apr 18;25(1):58. doi: 10.1186/s10194-024-01772-9 (PMC11027425; doi:10.1186/s10194-024-01772-9)
Supplement: Supplementary file 1 — Supplementary Material 1 [file 10194_2024_1772_MOESM1_ESM.docx]

**Supplementary Table 1.** Estimated coefficients, coefficients’ standard error (SE), 95% CI and p-values for salivary CGRP quantification of the fitted GLMM during a headache attack characterization after 12 weeks of Erenumab.

| **Independent Variables^†^** | **Estimate** | **SE** | **95% CI** | **P-value^‡^** |
| --- | --- | --- | --- | --- |
| *(Intercept)* | 10.95 | 0.867 | 9.25 – 11.65 | **<0.001** |
| **Main effects** | | | | |
| Age, y | -0.993 | 0.393 | -1.76 – -0.224 | **0.011** |
| Depressive symptoms (at baseline)  No (Ref.)  Yes | -  4.76 | -  0.831 | -  3.13 – 6.39 | -  **<0.001** |
| Treated attack (acute medication)  No (Ref.)  Yes | -  -1.41 | -  1.15 | -  -3.66 – 0.848 | -  0.222 |
| Attack evolution  Linear (L)  Quadratic (Q) | 1.32  -0.949 | 4.03  3.97 | -6.58 – 9.23  -8.73 – 6.84 | 0.743  0.811 |
| Treatment period  Baseline (Ref.)  12-week follow-up | -  2.53 | -  0.862 | -  0.843 – 4.22 | -  **0.003** |
| **Two-way interactions** | | | | |
| DS × Treatment period | -5.52 | 1.20 | -7.87 – -3.17 | **<0.001** |
| DS × Attack evolution [L] | 0.301 | 4.49 | -8.49 – 9.09 | 0.946 |
| DS × Attack evolution [Q] | -0.493 | 4.45 | -9.21 – 8.22 | 0.912 |
| Attack evolution [L] × Treatment period | 1.95 | 4.63 | -7.13 – 11.03 | 0.674 |
| Attack evolution [Q] × Treatment period | -1.17 | 4.75 | -10.47­­ – 8.14 | 0.806 |
| **Three-way interactions** | | | | |
| DS × Treatment period × Attack evolution [L] | 1.72 | 6.04 | -10.12 – 13.57 | 0.776 |
| DS × Treatment period × Attack evolution [Q] | -2.04 | 6.12 | -14.04 – 9.95 | 0.738 |

SE: Standard Error; CI: confidence interval; L: linear; Q: quadratic; DS: presence of depressive symptoms; ‘×’ symbol indicates interaction between variables.

**Bold** font indicates statistically significant variables. †Continuous independent variables were rescaled to a z-score metric (mean = 0, SD = 1) in the mixed model; ‡Statistical significance assessed the analysis of Deviance in each model (Type III Wald chi-square test).

**Supplementary Figure 1.** Salivary CGRP quantification (pg/mL) during a migraine attack according to the presence of depressive symptoms at baseline and after 12-weeks of treatment with erenumab.

**
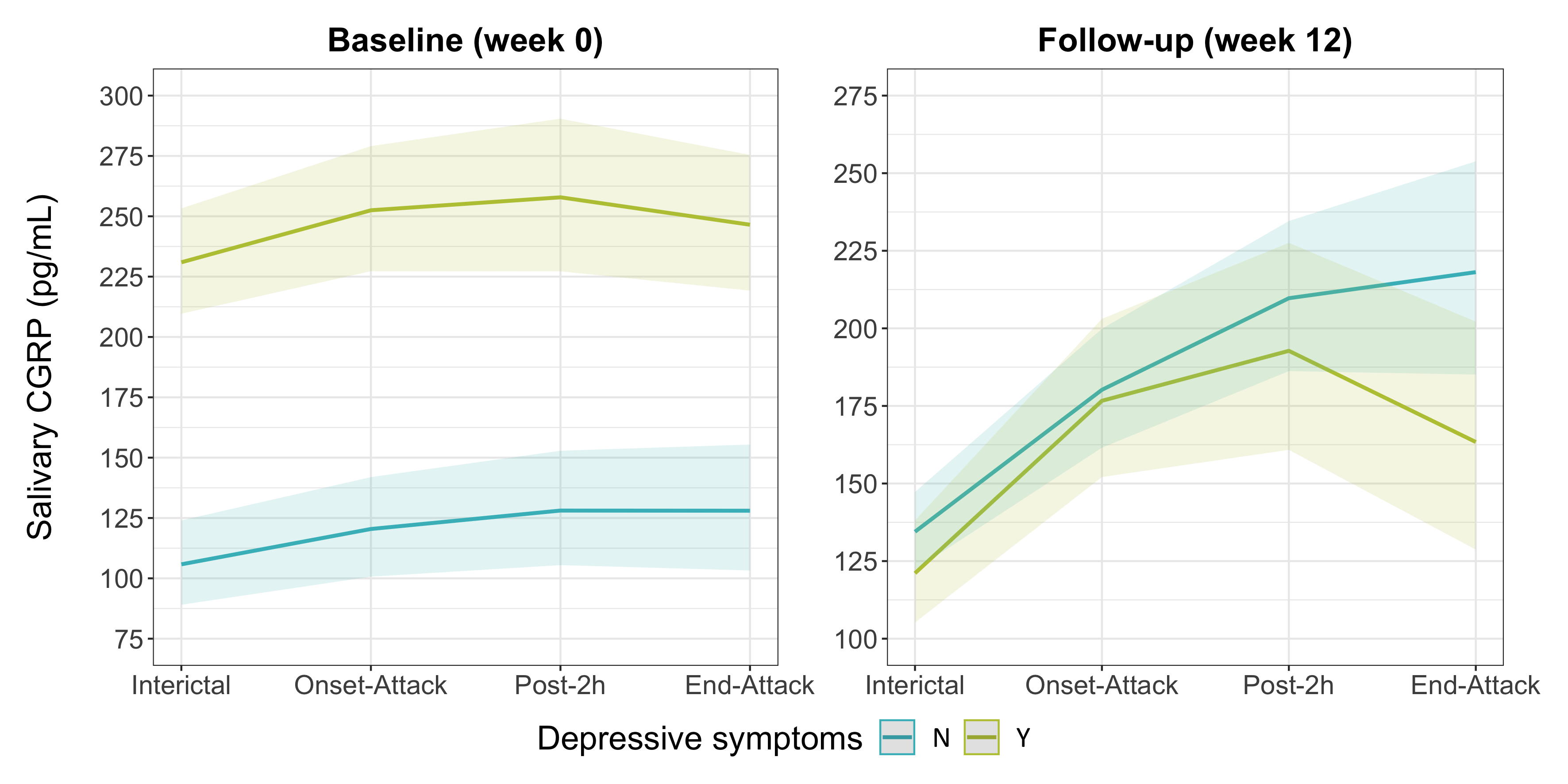
**

All predictors were rescaled to a z-score metric (mean = 0, SD = 1) in the prediction model. The shadow zone represents the 95% confidence levels of the GLMM estimation.
